# Increased breast cancer risk with HABP1/p32/gC1qR genetic polymorphism rs2285747 and its upregulation in northern Chinese women

**DOI:** 10.18632/oncotarget.14737

**Published:** 2017-01-19

**Authors:** Yongdong Jiang, Hao Wu, Jing Liu, Yanbo Chen, Jingjing Xie, Yashuang Zhao, Da Pang

**Affiliations:** ^1^ Department of Breast Surgery, Harbin Medical University Cancer Hospital, Harbin, China; ^2^ Department of Anesthesiology, The Second Affiliated Hospital, Harbin Medical University, Harbin, China; ^3^ Department of Epidemiology, Public Health College of Harbin Medical University, Harbin, China; ^4^ Sino-Russian Medical Research Center, Heilongjiang Academy of Medical Sciences, Harbin, China

**Keywords:** breast cancer, HABP1 gene, single nucleotide polymorphisms, protein expression, Chinese

## Abstract

**Object:**

Hyaluronic acid binding protein 1 (HABP1/p32/gC1qR) is overexpressed in breast cancer. However, it is unknown whether HABP1 gene polymorphisms affect breast cancer risk. This study aims to evaluate the potential association of single nucleotide polymorphisms (SNPs) of HABP1 with breast cancer in northern Chinese women.

**Results:**

The minor allele of rs2285747 was strongly associated with breast cancer with OR of 1.553 (95% CI = 1.251–1.927). SNP rs2285747 was also associated with high HABP1 protein expression under the co-dominant and dominant model (*p* = 0.005, *p* = 0.019, respectively). For rs2472614, the patients with CG and GG were more likely to have HER2 negative tumors compared to CC (*p* = 0.015). For rs3786054, the patients with AG and GG were more likely to have HER2 and P53 negative breast cancer compared to AA (*p* = 0.024, *p* = 0.064, receptively).

**Materials and Methods:**

Seven SNPs were analyzed in 505 breast cancer patients and 505 controls using SNaPshot method. The associations between SNPs and breast cancer were examined by logistic regression. The associations of SNPs with HABP1 protein expression and disease characteristics were examined by chi-square test.

**Conclusions:**

SNP rs2285747 of HABP1 increased breast cancer risk and elevated its protein expression in northern Chinese women.

## INTRODUCTION

Breast cancer is presently the most frequently occurring cancer in Chinese women as in most western countries [1]. Although its exact etiology remains elusive, accumulating evidences suggest that breast cancer result from a complex interaction of genetic, environmental and lifestyle factors [2, 3]. Many genes, such as high-penetrance susceptibility genes like BRCA1 [4] or BRCA2 [5] and low-penetrancce susceptibility genes [6], have been identified to be involved in breast tumorigenesis and progression. It is necessary to explore the potential mechanisms of these genes as effective molecular biomarkers in breast cancer.

Hyaluronic acid binding protein 1 (HABP1/p32/gC1qR) is an cell adhesion protein and receptor for hyaluronic acid (HA) [7, 8]. HABP1 belongs to the hyaladherin family and may play an important role in regulating cellular signaling. The HABP1 gene locates at human chromosome 17p12-13, including six exons and five introns, and is highly expressed in various tissues and cell types [9]. HABP1 is a multi-functional protein involved in the activation of the complement and kinin systems [10]. Accumulating data demonstrates that upregulation of HABP1 promotes tumorigenesis by enhancing the proliferation, invasion and metastasis of cancer cells [11–13]. Overexpression of HABP1 is observed in several cancers, such as lung, gastric, colon adenocarcinomas, epidermal carcinoma, ovarian cancer, and endometrial cancer [14, 15]. We previously found that the mRNA and protein levels of HABP1 significantly increased in breast cancer tissues, and upregulation of HABP1 was significantly associated with poor prognosis of breast cancer, including triple-negative breast cancer [12, 16]. These studies suggest that HABP1 may be a novel biomarker for the prognosis of breast cancer. However, the underlying molecular mechanism of the dysregulation of HABP1 in breast cancer is largely unknown. It is well known that the genetic polymorphisms could affect gene expression. Thus, we may hypothesize that the genetic variants of HABP1 may associate with mRNA and protein expression and affect breast cancer risk and prognosis.

In this study, we selected seven potential functional SNPs (rs1050390, rs1050461, rs2285747, rs2472614, rs3786054, rs4790264, and rs8072363) in the HABP1 gene from the dbSNP and HapMap databases using a combined analysis of functional significance and Tag SNP strategies, and performed genotyping analyses in 505 breast cancer patients and 505 healthy controls to investigate the associations of HABP1 gene polymorphisms with breast cancer susceptibility, the survival, the clinicopathological features and the HABP1 protein expression in a population from northeast China, Heilongjiang Province.

## RESULTS

### Subject characteristics

The characteristics of 505 breast cancer cases and 505 cancer-free controls were summarized in Table 1. There were significant differences between cases and controls in the BMI, age at menarche, and breastfeeding duration. Compared with controls, cases tended to have significantly higher BMI, older age at menarche and longer breastfeeding duration. Of all subjects, 96 (19.0%) cases and 70 (13.1%) controls reported a family history of cancer in first-degree relatives, which was significantly different between cases and controls (*p* < 0.05). Among 96 patients who had family history of cancer, 13 had family history of breast cancer. No one had family history of breast cancer among 70 controls.

**Table 1:** Distribution of selected variables in breast cancer cases and cancer-free controls

Variables	Cases, *n* = 505	Controls, *n* = 505	*p* value
Age (year)	49.40 ± 10.34 (49.00)	49.43 ± 9.92 (49.00)	0.970
Body mass index (kg/m)^2^, BMI	24.29 ± 3.41 (24.09)	23.11 ± 2.84 (23.05)	< 0.001
Age at menarche (year)	15.42 ± 1.79 (15.00)	15.06 ± 1.85 (15.00)	0.002
Age at first live birth (year)	23.96 ± 6.09 (25.00)	24.44 ± 6.99 (26.00)	0.230
Age at menopause (year)	49.37 ± 3.88 (50.00)	49.98 ± 4.08 (50.00)	0.107
Menopausal status			0.526
Pre-menopausal	289 (56.8)	279 (55.2)	
Post-menopausal	216 (57.2)	226 (44.8)	
Breastfeeding duration (months)	16.03 ± 13.13 (12.00)	11.53 ± 7.51(12.00)	< 0.001
Family history of cancer			0.027
Positive	96 (19.0)	70 (13.1)	
Negative	409 (81.0)	435 (86.9)	

Note: Data presented as the mean ± standard deviation (median) or number (% of total number).

### Associations between SNPs and breast cancer risk

The genotype distributions of HABP1 rs1050390, rs1050461, rs2285747, rs2472614, rs3786054, rs4790264 and rs8072363 and their associations of SNP with breast cancer risk were summarized in Table 2. The observed genotype frequencies of seven SNPs followed Hardy-Weinberg equilibrium among the controls (*p* > 0.05 for all seven SNPs). In the logistic regression models, compared with CC genotype of rs2285747, CG and GG genotypes were associated with an increased risk of breast cancer (adjusted OR = 1.619, 95% CI = 1.232–2.129 for CG; OR = 2.151, 95% CI = 1.198–3.863 for GG, respectively) (Table 2). This SNP was also associated with an increased risk of breast cancer under a dominant model (GG+CG vs. CC, OR = 1.683, 95% CI = 1.295–2.186). However, no significant association with breast cancer risk was observed for other six SNPs in the HABP1 gene.

**Table 2:** The associations between HABP1 gene polymorphisms and breast cancer risk

SNP	Genotype	Cases *n* = 505 (%)	Controls *n* = 505 (%)	OR (95% CI)^a^	*p* value
rs1050390	AA	300 (59.4)	304 (60.2)	1	
	AG	186 (36.8)	182 (36.0)	1.088 (0.833–1.422)	0.536
	GG	19 (3.8)	19 (3.8)	1.085 (0.554–2.125)	0.812
	AG + GG	205 (40.6)	201 (39.8)	1.088 (0.839–1.411)	0.525
	G^b^	22.2	21.8	1.066 (0.858–1.324)	0.563
rs1050461	CC	301 (59.6)	304 (60.2)	1	
	CT	185 (36.6)	182 (36.0)	1.075 (0.823–1.405)	0.596
	TT	19 (3.8)	19 (3.8)	1.080 (0.551–2.114)	0.823
	CT + TT	204 (40.4)	201 (39.8)	1.076 (0.829–1.395)	0.583
	T^b^	22.1	21.8	1.314 (1.058–0.851)	0.613
rs2285747	CC	273 (54.1)	332 (65.7)	1	
	CG	196 (38.8)	153 (30.3)	1.619 (1.232–2.129)	0.001
	GG	36 (7.1)	20 (4.0)	2.151 (1.198–3.863)	0.010
	CG + GG	232 (45.9)	173 (34.3)	1.683 (1.295–2.186)	< 0.001
	Gb	26.5	19.1	1.553 (1.251–1.927)	< 0.001
rs2472614	CC	203 (40.2)	205 (40.6)	1	
	CG	234 (46.3)	230 (45.5)	1.082 (0.822–1.424)	0.575
	GG	68 (13.5)	70 (13.9)	0.939 (0.630–1.398)	0.756
	CG + GG	302 (59.8)	300 (59.4)	1.047 (0.808–1.357)	0.729
	G^b^	36.6	36.6	0.997 (0.828–1.201)	0.977
rs3786054	AA	195 (38.6)	196 (38.8)	1	
	AG	235 (46.5)	236 (46.7)	1.048 (0.794–1.382)	0.742
	GG	75 (14.9)	73 (14.5)	0.995 (0.674–1.469)	0.980
	AG + GG	310 (61.4)	309 (61.2)	1.035 (0.797–1.344)	0.798
	G^b^	38.1	37.8	1.009 (0.839–1.214)	0.923
rs4790264	TT	300 (59.4)	304 (60.2)	1	
	TG	186 (36.8)	182 (36.0)	1.088 (0.833–1.422)	0.536
	GG	19 (3.8)	19 (3.8)	1.085 (0.554–2.125)	0.812
	TG + GG	205 (40.6)	201 (39.8)	1.088 (0.839–1.411)	0.525
	G^b^	22.2	21.8	1.066 (0.858–1.324)	0.563
rs8072363	TT	300 (59.4)	304 (60.2)	1	
	TC	186 (36.8)	183 (36.2)	1.088 (0.833–1.422)	0.536
	CC	19 (3.8)	18 (3.6)	1.158 (0.586–2.290)	0.673
	TC + CC	205(40.6)	201(39.8)	1.094(0.844–1.419)	0.497
	C^b^	22.2	21.7	1.078 (0.867–1.339)	0.500

^a^Adjusted for age, BMI, age at menarche, menopausal status, and family history of cancer. ^b^minor allele frequency.

### Association of SNPs with HABP1 protein expression

**Figure 1:**
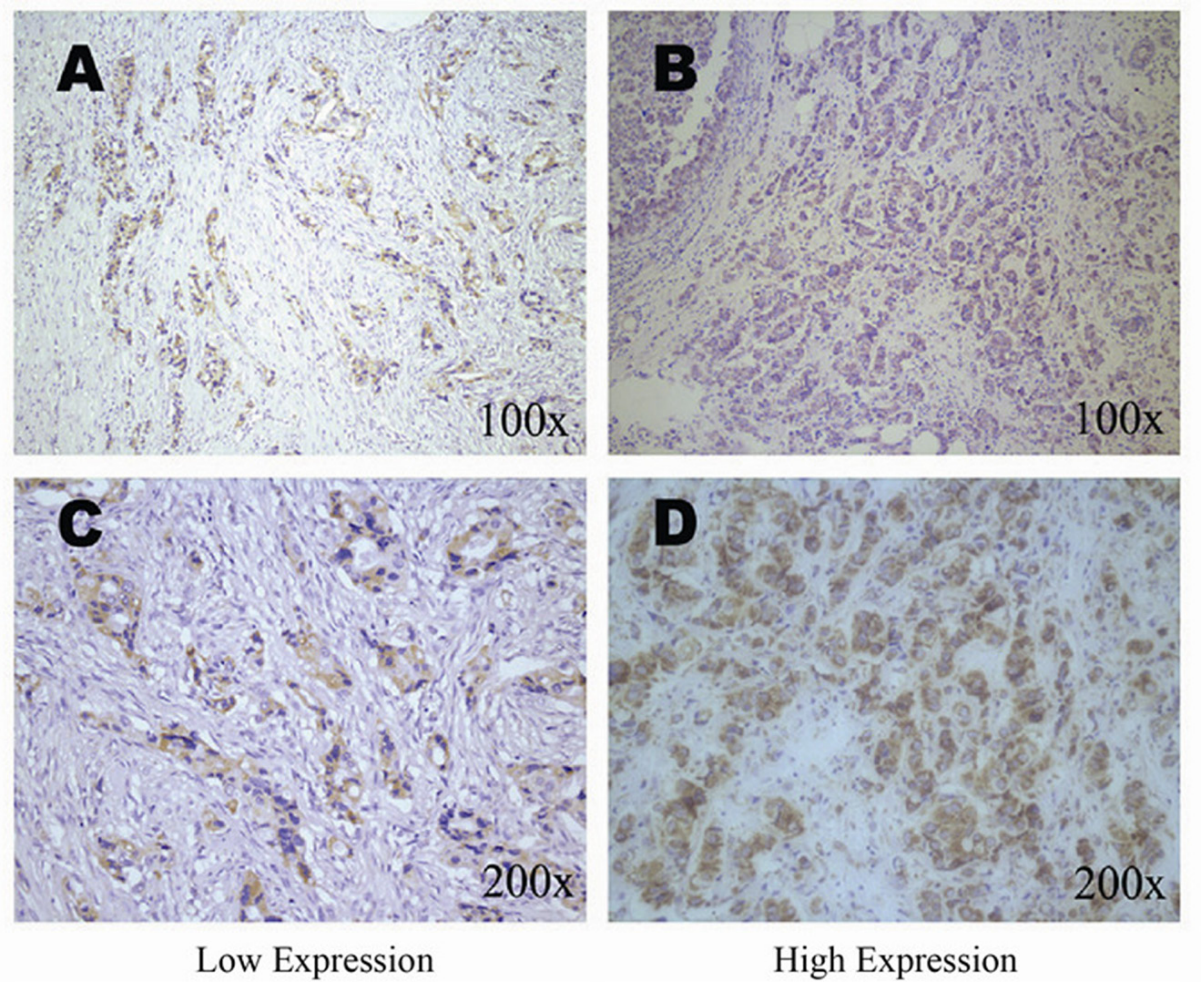
Immunohistochemical staining of HABP1 in breast tissues HABP1 immunoreactivity was observed mainly in the cytoplasm. Staining for each specimen is shown at two magnification: top, 100×; bottom, 200×. HABP1 protein low expression specimens (A, C); HABP1 protein low expression specimens (B, D).

We analyzed the correlations between HABP1 gene polymorphisms and protein expression (Table 3). HABP1 protein expression was shown in 390 breast cancer tissues. In the 10 cases of ductal carcinoma, 6 cases were low expression, and 4 cases were high expression. In the 359 cases of invasive ductal carcinoma, 133 cases were low expression, and 226 cases were high expression. In the other types, 10 cases were low expression, and 11 cases were high expression. The HABP1 protein expression in breast cancer tissue was shown in Figure 1, and the staining were localized within the cytoplasm. Under the co-dominant model, we found that the SNP rs228547 was significantly associated with higher/elevated HABP1 protein expression (*p* = 0.005). Moreover, the patients with genotypes CG and GG were associated with high HABP1 protein expression under the dominant model (*p* = 0.019). There were no significant associations between the other six SNPs and HABP1 protein expression under either codominant or dominant model (*p* > 0.05).

**Table 3:** Association of HABP1 genetic polymorphisms with HABP1 protein expression

SNP	Genotype	NO.	HABP1 protein expression		*p* value
			Low (%)	High (%)	
rs1050390	AA	216	90 (60.4%)	126 (52.3%)	
	AG	159	55 (36.9%)	104 (43.2%)	
	GG	15	4 (2.7%)	11 (4.6%)	0.244
	AG + GG vs AA	174	59 (39.6%)	115 (47.8%)	0.117
rs1050461	CC	217	90 (60.4%)	127 (52.7%)	
	CT	158	55 (36.9%)	103 (42.7%)	
	TT	15	4 (2.7%)	11 (4.6%)	0.273
	CT + TT vs CC	173	59(39.6%)	114 (47.3%)	0.137
rs2285747	CC	195	88 (59.1%)	107 (44.4%)	
	CG	163	51 (34.2%)	112 (46.5%)	
	GG	32	10 (6.7%)	22 (9.1%)	0.019
	CG + GG vs CC	195	61 (40.9%)	134 (55.6%)	0.005
rs2472614	CC	151	65 (43.6%)	86 (35.7%)	
	CG	185	69 (46.3%)	116 (48.1%)	
	GG	54	15 (10.1%)	39 (16.2%)	0.132
	CG + GG vs CC	239	84 (56.4%)	155 (64.3%)	0.118
rs3786054	AA	145	64 (43.0%)	81 (33.6%)	
	AG	184	66 (44.3%)	118 (49.0%)	
	GG	61	19 (12.8%)	42 (17.4%)	0.144
	AG + GG vs AA	245	85 (57.1%)	160 (66.4%)	0.064
rs4790264	TT	216	90 (60.4%)	126 (52.3%)	
	TG	159	55 (36.9%)	104 (43.2%)	
	GG	15	4 (2.7%)	11 (4.6%)	0.244
	TG + GG vs TT	174	59 (39.6%)	115 (47.8%)	0.117
rs8072363	TT	217	90 (60.4%)	127 (52.7%)	
	CT	158	55 (36.9%)	103 (42.7%)	
	CC	15	4 (2.7%)	11 (4.6%)	0.273
	CT + CC vs TT	173	59 (39.6%)	114 (47.3%)	0.137

### Associations between SNPs and the clinicopathological features of breast cancer

We next analyzed the effects of the seven SNPs in HABP1 gene on a series of clinicopathological features in the patient cohort, including clinic stage, tumor size, Bloom-Richardson grade, lymph node metastasis and the expressions of ER, PR, HER2 (also named as c-erbB-2), Ki67 and P53. The clinicopathologic features of breast cancer patients were shown in Table 4.

**Table 4:** Summary of the clinicopathologic features of breast cancer studied

Variables		NO.(Percentage)
Clinic stage	0	14 (2.77)
	I	112 (22.18)
	II	214 (42.38)
	III–IV	115 (22.77)
	Unknown	50 (9.90)
Tumor size (cm)	≤ 2	178 (35.25)
	> 2	267 (52.87)
	Unknown	60 (11.88)
Bloom-Richardson grade	1	35 (6.93)
	2	276 (54.65)
	3	125 (24.75)
	Unknown	69 (13.66)
Tumor type	DCIS	16 (3.16)
	IDC	454 (89.90)
	Others	35 (6.93)
LN involvement	Negative	275 (54.46)
	Positive	214 (42.38)
	Unknown	16 (3.17)
ER	Negative	190 (37.62)
	Positive	312 (61.78)
	Unknown	3 (0.59)
PR	Negative	227 (44.95)
	Positive	275 (54.46)
	Unknown	3 (0.59)
HER2	Negative	361 (71.49)
	Positive	29 (5.74)
	Unknown	115 (22.77)
Ki67	≤ 14%	191 (37.82)
	> 14%	311 (61.58)
	Unknown	3 (0.59)
P53	Negative	389 (77.03)
	Positive	113 (22.38)
	Unknown	3 (0.59)
HABP1 protein expression	Low	149 (29.51)
	High	241 (47.72)
	Unknown	115 (22.77)

Note: DCIS: ductal carcinoma in situ; IDC: infitrating ductal carcinoma.

For rs2472614, it was found that the patients with genotype CG and GG were more likely to have HER2 negative tumors compared to the patients with genotype CC (*p* = 0.015). And for SNP rs3786054, the patients with genotype AG and GG were more likely to have HER2 and P53 negative breast cancer relative to the patients with genotype AA (*p* = 0.024 and *p* = 0.064, receptively). In this research, for SNP rs1050390, rs1050461, rs2285747, rs4790264 and rs8072363, we did not find the significant association between these SNPs and the clinicopathological features (*p* > 0.05) (Supplementary Tables 1–7).

### HABP1 expression is correlated with breast cancer patient survival

**Figure 2:**
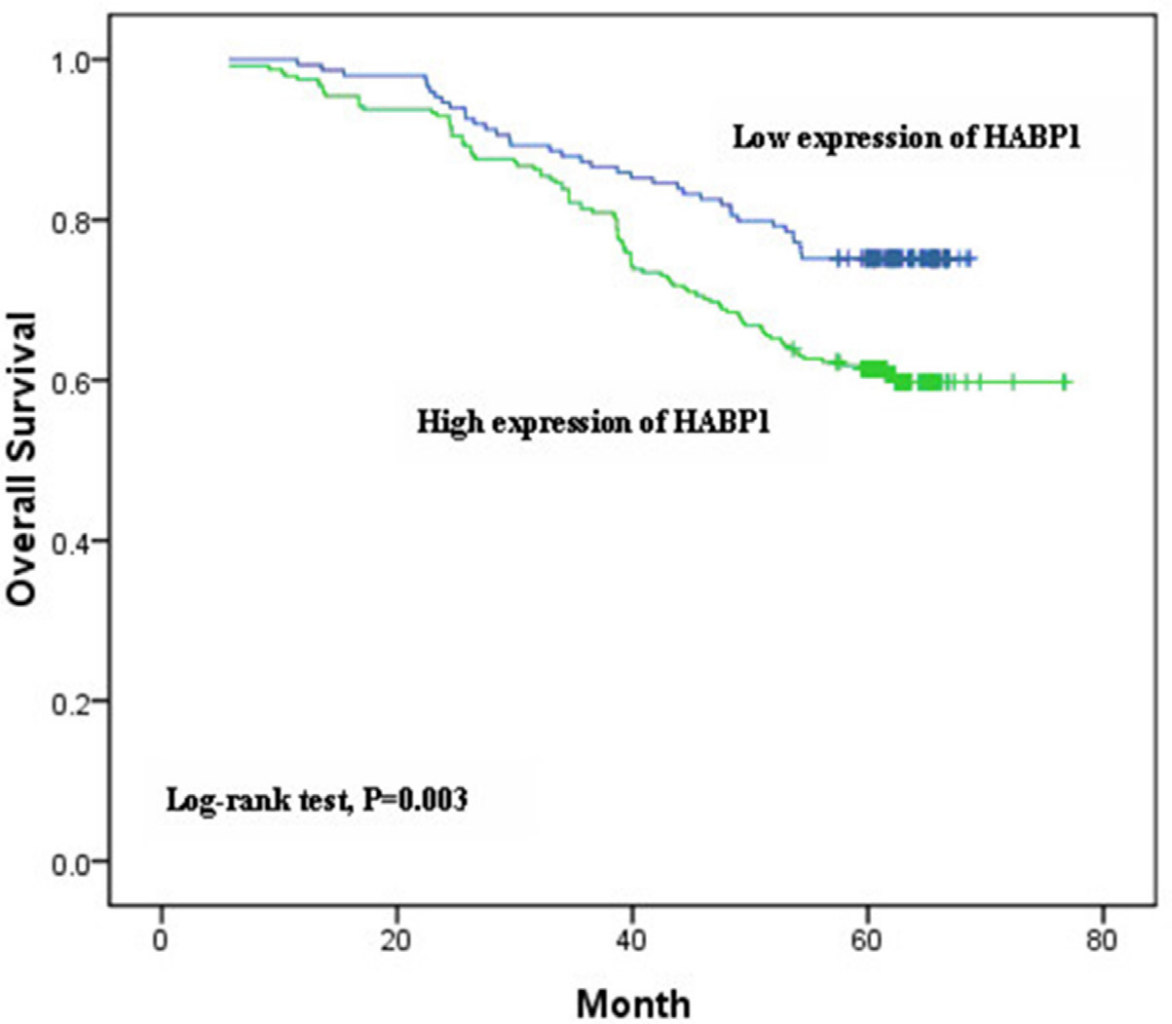
Kaplan-Meier survival analyses of breast cancer patients

We then analyzed the associations of overall survival (OS) with seven SNPs in HABP1 gene, HABP1 protein expression, and clinicopathological features. Among 505 breast cancer patients, 41 was loss of follow up, and 323 remained alive after the expiration of the follow-up period. The OS rate was 69.6%. We found that the clinic stage (*p* = 7.201 × 10^−7^), Bloom-Richardson grade (1 vs 3: *p* = 0.030; 2 vs 3: *p* = 0.010), the status of LN involvement (*p* = 0.001), ER (*p* = 4.579 × 10^−4^), PR (*p* = 0.001), HER2 (*p* = 0.020), P53 (*p* = 0.024), HABP1 protein expression (*p* = 0.004), and rs1050390 (*p* = 0.039), rs4790264 (*p* = 0.039), rs8072363 (*p* = 0.033) were associated with the OS under univariate analysis (Table 5). Under multivariate analysis (Table 5), the clinic stage (*p* = 0.023), the expression of ER (*p* = 1.019 × 10^−4^) and HABP1 protein (*p* = 0.005) were associated with the OS. Kaplan-Meier analysis shown that HABP1 expression was significantly related to OS (log-rank test, *p* = 0.003; Figure 2).

**Table 5:** Univariate and multivariate analysis for overall survival in breast cancer patients

Variables	Univariate analysis		Multivariate analysis	
	OR (95% CI)	*p* value	OR (95% CI)	*p* value
Clinic stage (0, I, II/III–IV)	2.369 (1.684–3.332)	7.201 × 10^−7^	1.714 (1.076–2.729)	0.023
Tumor size (≤ 2/> 2)	1.391 (0.971−1.993)	0.072		
Bloom-Richardson grade (1/2)	3.040 (1.112−8.311)	0.03		
Bloom-Richardson grade (1/3)	3.870 (1.390−10.773)	0.01		
LN involvement (Negative/Positive)	1.790 (1.276−2.510)	0.001		
ER (Negative/Positive)	0.553 (0.397−0.770)	4.579 × 10^−4^	0.402 (0.254–0.637)	1.019 × 10^−4^
PR (Negative/Positive)	0.572 (0.409−0.798)	0.001		
HER2 (Negative/Positive)	2.052 (1.119−3.765)	0.02		
Ki67 (≤ 14%/> 14%)	1.218 (0.856−1.734)	0.273		
P53 (Negative/Positive)	1.522 (1.057−2.190)	0.024		
HABP1protein expression (Low/High)	1.756 (1.201−2.568)	0.004	2.235 (1.282–3.896)	0.005
rs1050390 (AA/AG)	1.425 (1.016−1.999)	0.04		
rs1050390 (AA/GG)	1.336 (0.581−3.073)	0.495		
rs1050390 (AA/AG + GG)	0.706 (0.507−0.982)	0.039		
rs1050461 (CC/CT)	1.383 (0.985−1.941)	0.061		
rs1050461 (CC /TT)	1.318 (0.574−3.030)	0.515		
rs1050461 (CC/CT + TT)	1.377 (0.989−1.916)	0.058		
rs2285747 (CC/CG)	1.360 (0.963−1.920)	0.081		
rs2285747 (CC/GG)	1.372 (0.741−2.538)	0.314		
rs2285747 (CC/CG + GG)	1.362 (0.978−1.896)	0.067		
rs2472614 (CC/CG)	1.202 (0.837−1.727)	0.318		
rs2472614 (CC/GG)	1.182 (0.704−1.986)	0.527		
rs2472614 (CC/CG + GG)	1.198 (0.848−1.692)	0.305		
rs3786054 (AA/AG)	1.333 (0.922−1.926)	0.126		
rs3786054 (AA/GG)	1.150 (0.686−1.927)	0.596		
rs3786054 (AA/AG + GG)	1.287 (0.905−1.831)	0.16		
rs4790264 (TT/TG)	1.425 (1.016−1.999)	0.04		
rs4790264 (TT/GG)	1.336 (0.581−3.073)	0.495		
rs4790264 (TT/TG + GG)	1.416 (1.018−1.971)	0.039		
rs8072363 (TT/TC)	1.441 (1.027−2.021)	0.035		
rs8072363 (TT/CC)	1.342 (0.584−3.085)	0.488		
rs8072363 (TT/TC + CC)	1.431 (1.029−1.992)	0.033		

Note: (A/B), A is the reference.

## DISCUSSION

In this study, we found that SNP rs2285747 in the HABP1 gene could increase breast cancer risk and elevate HABP1 protein expression in our population. Kaplan-Meier analysis revealed that HABP1 expression was significantly associated with poor prognosis in breast cancer patients, univariate and multivariate analyses indicated that HABP1 expression was an independent prognostic factor. It suggested that HABP1 gene polymorphisms might increase breast cancer risk by affecting its protein expression and result in patient's unfavourable prognosis. To the best of our knowledge, it is the first study to evaluate the relations between HABP1 genetic polymorphisms and breast cancer risk in a large cohort of Chinese northern women patients.

Our previous study showed that high HABP1 mRNA expression in breast cancer indicated poor prognosis and the 5-year survival rate was much lower in the high expression group compared with the low ones [12]. And we also found that HABP1 protein expression was an independent prognostic factor for breast cancer survival [17], especially for triple-negative breast cancer [16]. In the present study, we found that among the seven potential functional SNPs of the HABP1 gene, SNP rs2285747 was significantly associated with breast cancer risk and HABP1 protein expression in Chinese women of Heilongjiang Province. The carriers with CG and GG genotypes of rs2285747 had a higher risk of breast cancer and HABP1 protein expression. These results suggested that genetic variants in the HABP1 gene might associate with mRNA and protein expression of HABP1 and increase breast cancer susceptibility. SNP rs2285747 locates at the intron of HABP1 gene. The global MAF in the PUBMED was 0.390. In the disease associated with intron, most of the genetic mutations are concentrated in the intron and exon junction. These mutuations could cause exon deletion or intron not being cut. And the variation in the middle position of intron could cause disease due to activation of the recessive splice sites and the change of shear effect [18]. In our study, we found that SNP rs2285747 was not associated with OS in survival analysis. But we found that the clinic stage, the expression of ER and HABP1 protein were associated with OS under multivariate analysis.

SNP rs2472614, rs3786054, rs4790264, and rs8072363, located at the introns of HABP1 gene, were not associated with breast cancer risk in our study. Their global MAF in the PUBMED was 0.263, 0.314, 0.389 and 0.390, respectively. We found that SNP rs1050390 and rs1050461, located at the 3′ UTR of HABP1 gene, were not correlated with breast cancer risk in our population. Their global MAF in the PUBMED was 0.381 and 0.396, respectively.

Our studies also indicated that SNP rs2472614 and rs3786054 were significantly associated with HER2 negative breast cancer. Since HER2 is overexpressed at the cell surface of tumor cells, and it might predict an unfavourable prognosis. But we did not find that the two SNPs were associated with OS in the survival analysis. So, the genotype of SNP rs2472614 and rs3786054 need further to be validated in a larger population.

The mechanism of the promotion of tumorigenesis by HABP1 is currently largely unknown. It was reported that HABP1 was a receptor for the tumor homing peptide Lyp1 which specifically recognized an epitope in tumor lymphatics and tumor cells in certain cancers [13]. Knocking down HABP1 inhibited the growth of tumor cells [19]. In contrast, overexpression of HABP1 disturbed normal cell polarization and ultimately led to the malignant transformation of normal cells [20]. However, DNA sequencing of the gene encoding tumor-associated HABP1 did not reveal any consistent tumor specific mutations [21].

In conclusion, it is the first study to evaluate the association between HABP1 gene polymorphisms and breast cancer risk in women from Northeast China. Our results could provide a new idea that the mechanisms of HABP1 interfered with breast cancer, and suggest that HABP1 gene may be a potential biomarker for the early detection and prognosis, and a target for the development of molecular targeted therapy for breast cancer.

## MATERIALS AND METHODS

### Subjects

A cohort of 1,013 individuals, comprising 505 breast cancer patients and 508 healthy controls, were included in this study. Sporadic breast cancer patients were recruited from the Department of Breast Surgery, the Third Affiliated Hospital of Harbin Medical University. Breast cancer was diagnosed according to the patient's surgical and pathological analyses, and all the patients did not have radiotherapy or chemotherapy history before surgical operations. The control group was chosen from Han origin women living in Harbin, a province in the northeast of China. The control group was matched for age and ethnicity with patients, without a history of cancer. The participants were genetically not related in three generations. After providing informed consent, each participant was interviewed to collect detailed information on demographic characteristics (Table 1) and provided 5 ml venous blood from September 2008 to May 2009. The clinicopathologic features of breast cancer patients were shown in Table 4. The patients received a minimum of four courses of anthracycline-based and/or taxane-based chemotherapy after surgery. Hormone treatment with tamoxifen or aromatase inhibitors was given to the patients with hormone receptor positive (ER or PR, or both). Her-2-positive patients who agreed to receive anti-Her-2 targeted therapy were treated with adjuvant trastuzumab for 1 year. This study was approved by the ethics committee of Harbin Medical University.

### SNPs selection and genotyping

We performed a combined analysis of functional significance and Tag SNP strategies to select seven potential functional SNPs of the HABP1 gene from the dbSNP and HapMap databases. We selected tag SNPs from the entire length of the HABP1 gene (GRCh38.p7, chr17: 5,432,777-5,448,830) and additional 5kb of upstream and 2kb of downstream sequences to include the promoter and potential regulatory regions. The seven SNPs were rs1050390, rs1050461, rs2285747, rs2472614, rs3786054, rs4790264, and rs8072363, respectively. The minor allele frequency (MAF) of these SNPs was greater than 5%, or pair-wise r^2^ was more than 0.8. Genomic DNA was isolated from EDTA anti-coagulated whole blood using the AxyPrep Blood Genomic DNA Miniprep Kit (Axygen Biotechnology, Union City, CA, USA). The SNaPshot SNP assay was carried out to detect the dimorphism at the seven SNP loci. The resulting data were analyzed with GeneMapperTM 4.0 Software (Applied Biosystems, Foster City, CA, USA). The reaction conditions and protocol for the genotyping were shown in Supplementary Materials and primer/ probe sequences were shown in Supplementary Table 8. To ensure quality-control, genotyping was done without knowledge of case/control status of the subjects, and a 5% random sample of cases and controls was genotyped twice by different persons; the reproducibility was 100%. Genotyping failed in three controls due to DNA quality and the average call rate for all SNPs was higher than 99%. All data from these 3 women were excluded from analyses. Therefore, 505 breast cancer cases and 505 controls were included in the final analyses.

### Immunohistochemistry

The formalin-fixed, paraffin-embedded samples were cut into 4μm and stained with H&E for tumor confirmation. The tissue sections were dried at 70°C for 3 h. After deparaffinization and hydration according to the standard procedures, sections were washed in phosphate-buffered saline (PBS; 3 × 3 min). The washed sections were treated with 3% H_2_O_2_ for 15min in the dark. After washing in distilled water, sections were washed in PBS (3 × 5 min) and were then treated with 0.01 mol/L citrate buffer (pH 6.0) and were exposed to heat induced epitope retrieval for 2 min. The sections were incubated overnight at 4°C with primary antibody HABP1 (1:200 dilution, a recombinant rabbit monoclonal antibody, Abcam: ab131284). After washing in PBS (3 × 5 min), each section was incubated with the secondary antibody (an anti-rabbit antibody, ZSGB-BIO: PV6001, K156605C) at room temperature for 30 min. After washing in PBS (3 × 5 min), each section was treated with diaminobenzadine (DAB: ZSGB-BIO: ZLI-9018, K152317J) working solution at room temperature for 3 min, and then washed in distilled water.

The immunohistochemical staining of HABP1 were scored by combining the proportion and intensity of positively stained tumor cells. The staining intensity was classified as 0 (no staining), 1 (weak staining), 2 (moderate staining), and 3 (strong staining). The percentage of positive cells was classified as 0 (no positive tumor cells), 1 (≤ 10% positive tumor cells), 2 (10–50% positive tumor cells), and 3 (≥ 50% positive tumor cells). Staining index (SI) was calculated as a proportion score × staining intensity score. The final scores ≤ 4 were considered to be low expression, and the remainder were classified as high expression. Cases with discrepancies were re-reviewed simultaneously by the original two pathologists (NXM and SHT) and a senior pathologist (LXM) until a consensus was reached.

### Follow-up

Patients were followed regularly for 5 years at the Third Affiliated Hospital of the Harbin Medical University. The follow-up period was 6 months during the first 2 year, and the follow-up period was 12 months during the next 3 year. 464 participants were followed up and 41 patients were excluded. The participation rate is 91.88%. Patients were followed up from the end of the treatment every year by the ways of the annual outpatient follow-up system, telephone and letters. Clinical records were obtained from the follow-up department of the hospital. Survival was calculated in months from the date of diagnosis to whichever of the following occurred first: the date of death, the date last known to be alive, or the follow-up cut-off date (June 1, 2014) in our study. The causes of death of breast cancer patients were breast cancer or others.

### Statistical analysis

The genotype frequencies were tested for Hardy–Weinberg equilibrium using the chi-square test among the controls. Differences between cases and controls in demographic characteristics were evaluated by the chi-square test (for categorical variables) or Student's *t-test* (for continuous variables). Associations between genotypes and breast cancer risk were estimated by computing odds ratios (ORs) and 95% confidence intervals (CIs) from logistic regression with adjustment for age, BMI, age at menarche, menopausal state, and family history of cancer. Homozygotes for non-risk alleles were the reference group. Heterozygotes and homozygous risk allele genotypes were compared with the reference group, respectively. The dominant model was analyzed with homozygote for risk allele and heterozygote versus the reference group. The Kaplan–Meier survival curves and log rank tests were used for survival curves. The Cox proportional hazards model was used to estimate the independent prognostic factors for OS. The Pearson's chi-square test was used to evaluate the correlations between SNPs of HABP1 gene and the HABP1 protein expression. All statistical tests were two-sided, and a *p value* equal to or less than 0.05 was considered statistically significant and a *p value* less than 0.1 was considered a possible trend that could be explored further in larger study groups. Statistical analyses were performed using SPSS for Windows software (version 16.0; SPSS, Chicago, IL, USA).

## SUPPLEMENTARY MATERIALS FIGURES AND TABLES


