# Supplementary material for: Increased breast cancer risk with HABP1/p32/gC1qR genetic polymorphism rs2285747 and its upregulation in northern Chinese women
Source: Oncotarget. 2017 Jan 19;8(8):13932–41. doi: 10.18632/oncotarget.14737 (PMC5355151; doi:10.18632/oncotarget.14737)
Supplement: Supplementary file 1 [file oncotarget-08-13932-s001.pdf]

Increased breast cancer risk with HABP1/p32/gC1qR genetic polymorphism rs2285747 and its upregulation in northern Chinese women

Supplementary Materials

Supplementary Table 1: Associations between rs1050390 and the clinicopathological features

clinicopathological features	NO.	AA (%)	AG (%)	GG (%)	*p* value	AG + GG (%)	*p* value
Clinic stage(UICC)							
0	14	8 (3.0%)	6 (3.5%)	0 (0.0%)		6 (3.2%)	
I	112	77 (28.6%)	32 (18.5%)	3 (23.1%)		35 (18.8%)	
II	214	121 (45.0%)	85 (49.1%)	8 (61.5%)		93 (50.0%)	
III–IV	115	63 (23.4%)	50 (28.9%)	2 (15.4%)	0.265	52 (28.0%)	0.122
Tumor size (cm)							
≤ 2	178	107 (40.7%)	67 (39.6%)	4 (30.8%)		71 (39.0%)	
> 2	267	156 (59.3%)	102 (60.4%)	9 (69.2%)	0.770	111 (61.0%)	0.723
Bloom-Richardson grade							
1 or 2	311	177 (69.1%)	119 (72.6%)	15 (93.8%)		134 (74.4%)	
3	125	79 (30.9%)	45 (27.4%)	1 (6.2%)	0.098	46 (25.6%)	0.228
LN involvement							
Negative	275	169 (58.3%)	93 (51.1%)	13 (76.5%)		106 (53.3%)	
Positive	214	121 (41.7%)	89 (48.9%)	4 (23.5%)	0.072	93 (46.7%)	0.273
ER							
Negative	190	112 (37.6%)	68 (36.6%)	10 (55.6%)		78 (38.2%)	
Positive	312	186 (62.4%)	118 (63.4%)	8 (44.4%)	0.281	126 (61.8%)	0.883
PR							
Negative	227	134 (45.0%)	82 (44.1%)	11 (61.1%)		93 (45.6%)	
Positive	275	164 (55.0%)	104 (55.9%)	7 (38.9%)	0.379	111 (54.4%)	0.891
HER2							
Negative	361	218 (92.0%)	129 (94.2%)	14 (87.5%)		143 (93.5%)	
Positive	29	19 (8.0%)	8 (5.8%)	2 (12.5%)	0.543	10 (6.5%)	0.586
Ki67							
≤ 14%	191	118 (39.6%)	68 (36.6%)	5 (27.8%)		73 (35.8%)	
> 14%	311	180 (60.4%)	118 (63.4%)	13 (72.2%)	0.526	131 (64.2%)	0.387
P53							
Negative	389	234 (78.5%)	142 (76.3%)	13 (72.2%)		155 (76.0%)	
Positive	113	64 (21.5%)	44 (23.7%)	5 (27.8%)	0.738	49 (24.0%)	0.503

Supplementary Table 2: Associations between rs1050461 and the clinicopathological features

clinicopathological features	NO.	CC (%)	CT (%)	TT (%)	*p* value	CT + TT (%)	*p* value
Clinic stage(UICC)							
0	14	8 (3.0%)	6 (3.5%)	0 (0.0%)		6 (3.2%)	
I	112	77 (28.5%)	32 (18.6%)	3 (23.1%)		35 (18.9%)	
II	214	122 (45.2%)	84 (48.8%)	8 (61.5%)		92 (49.7%)	
III–IV	115	63 (23.3%)	50 (29.1%)	2 (15.4%)	0.277	52 (28.1%)	0.132
Tumor size (cm)							
≤ 2	178	108 (40.9%)	66 (39.3%)	4 (30.8%)		70 (38.7%)	
> 2	267	156 (59.1%)	102 (60.7%)	9 (69.2%)	0.745	111 (61.3%)	0.636
Bloom-Richardson grade							
1 or 2	311	178 (69.3%)	118 (72.4%)	15 (93.8%)		133 (74.3%)	
3	125	79 (30.7%)	45 (27.6%)	1 (6.2%)	0.102	46 (25.7%)	0.252
LN involvement							
Negative	275	169 (58.1%)	93 (51.4%)	13 (76.5%)		106 (53.5%)	
Positive	214	122 (41.9%)	88 (48.6%)	4 (23.5%)	0.084	92 (46.5%)	0.320
ER							
Negative	190	113 (37.8%)	67 (36.2%)	10 (55.6%)		77 (37.9%)	
Positive	312	186 (62.2%)	118 (63.8%)	8 (44.4%)	0.271	126 (62.1%)	0.975
PR							
Negative	227	135 (45.2%)	81 (43.8%)	11 (61.1%)		92 (45.3%)	
Positive	275	164 (54.8%)	104 (56.2%)	7 (38.9%)	0.370	111 (54.7%)	0.970
HER2							
Negative	361	218 (91.6%)	129 (94.9%)	14 (87.5%)		143 (94.1%)	
Positive	29	20 (8.4%)	7 (5.1%)	2 (12.5%)	0.376	9 (5.9%)	0.362
Ki67							
≤ 14%	191	118 (39.5%)	68 (36.8%)	5 (27.8%)		73 (36.0%)	
> 14%	311	181 (60.5%)	117 (63.2%)	13 (72.2%)	0.551	130 (64.0%)	0.427
P53							
Negative	389	234 (78.3%)	142 (76.8%)	13 (72.2%)		155 (76.4%)	
Positive	113	65 (21.7%)	43 (23.2%)	5 (27.8%)	0.800	48 (23.6%)	0.616

Supplementary Table 3: Associations between rs2285747 and the clinicopathological features

clinicopathological features	NO.	CC (%)	CG (%)	GG (%)	*p* value	CG + GG (%)	*p* value
Clinic stage(UICC)							
0	14	8 (3.3%)	5 (2.8%)	1 (3.3%)		6 (2.8%)	
I	112	68 (27.9%)	36 (19.9%)	8 (26.7%)		44 (20.9%)	
II	214	108 (44.3%)	93 (51.4%)	13 (43.3%)		106 (50.2%)	
III–IV	115	60 (24.6%)	47 (26.0%)	8 (26.7%)	0.656	55 (26.1%)	0.349
Tumor size (cm)							
≤ 2	178	95 (39.9%)	72 (40.2%)	11 (39.3%)		83 (40.1%)	
> 2	267	143 (60.1%)	107 (59.8%)	17 (60.7%)	0.995	124 (59.9%)	0.969
Bloom-Richardson grade							
1 or 2	311	161 (69.1%)	127 (72.6%)	23 (82.1%)		150 (73.9%)	
3	125	72 (30.9%)	48 (27.4%)	5 (17.9%)	0.317	53 (26.1%)	0.270
LN involvement							
Negative	275	152 (57.6%)	102 (53.4%)	21 (61.8%)		123 (54.7%)	
Positive	214	112 (42.4%)	89 (46.6%)	13 (38.2%)	0.539	102 (45.3%)	0.518
ER							
Negative	190	101 (37.3%)	72 (36.7%)	17 (48.6%)		89 (38.5%)	
Positive	312	170 (62.7%)	124 (63.3%)	18 (51.4%)	0.396	142 (61.5%)	0.772
PR							
Negative	227	123 (45.4%)	86 (43.9%)	18 (51.4%)		104 (45.0%)	
Positive	275	148 (54.6%)	110 (56.1%)	17 (48.6%)	0.708	127 (55.0%)	0.935
HER2							
Negative	361	196 (91.6%)	143 (95.3%)	22 (84.6%)		165 (93.8%)	
Positive	29	18 (8.4%)	7 (4.7%)	4 (15.4%)	0.113	11 (6.2%)	0.418
Ki67							
≤ 14%	191	107 (39.5%)	75 (38.3%)	9 (25.7%)		84 (36.4%)	
> 14%	311	164 (60.5%)	121 (61.7%)	26 (74.3%)	0.287	147 (63.6%)	0.473
P53							
Negative	389	214 (79.0%)	151 (77.0%)	24 (68.6%)		175 (75.8%)	
Positive	113	57 (21.0%)	45 (23.0%)	11 (31.4%)	0.376	56 (24.2%)	0.391

Supplementary Table 4: Associations between rs2472614 and the clinicopathological features

clinicopathological features	NO.	CC (%)	CG (%)	GG (%)	*p* value	CG + GG (%)	*p* value
Clinic stage(UICC)							
0	14	5 (2.7%)	8 (3.8%)	1 (1.7%)		9 (3.3%)	
I	112	55 (29.6%)	45 (21.3%)	12 (20.7%)		57 (21.2%)	
II	214	80 (43.0%)	103 (48.8%)	31 (53.4%)		134 (49.8%)	
III–IV	115	46 (24.7%)	55 (26.1%)	14 (24.1%)	0.503	69 (25.7%)	0.220
Tumor size (cm)							
≤ 2	178	76 (41.8%)	75 (36.1%)	27 (49.1%)		102 (38.8%)	
> 2	267	106 (58.2%)	133 (63.9%)	28 (50.9%)	0.176	161 (61.2%)	0.529
Bloom-Richardson grade							
1 or 2	311	132 (73.3%)	136 (69.0%)	43 (72.9%)		179 (69.9%)	
3	125	48 (26.7%)	61 (31.0%)	16 (27.1%)	0.628	77 (30.1%)	0.438
LN involvement							
Negative	275	109 (55.3%)	130 (57.5%)	36 (54.5%)		166 (56.8%)	
Positive	241	88 (44.7%)	96 (42.5%)	30 (45.5%)	0.863	126 (43.2%)	0.740
ER							
Negative	190	72 (35.8%)	91 (38.9%)	27 (40.3%)		118 (39.2%)	
Positive	312	129 (64.2%)	143 (61.1%)	40 (59.7%)	0.730	183 (60.8%)	0.444
PR							
Negative	227	91 (45.3%)	107 (45.7%)	29 (43.3%)		136 (45.2%)	
Positive	275	110 (54.7%)	127 (54.3%)	38 (56.7%)	0.939	165 (54.8%)	0.984
HER2							
Negative	361	141 (88.7%)	174 (96.7%)	46 (90.2%)		220 (95.2%)	
Positive	29	18 (11.3%)	6 (3.3%)	5 (9.8%)	0.016	11 (4.8%)	0.015
Ki67							
≤ 14%	191	82 (40.8%)	88 (37.6%)	21 (31.3%)		109 (36.2%)	
> 14%	311	119 (59.2%)	146 (62.4%)	46 (68.7%)	0.379	192 (63.8%)	0.300
P53							
Negative	389	161 (80.1%)	176 (75.2%)	52 (77.6%)		228 (75.7%)	
Positive	113	40 (19.9%)	58 (24.8%)	15 (22.4%)	0.477	73 (24.3%)	0.253

Supplementary Table 5: Associations between rs3786054 and the clinicopathological features

clinicopathological features	NO.	AA (%)	AG (%)	GG (%)	*p* value	AG + GG (%)	*p* value
Clinic stage(UICC)							
0	14	6 (3.4%)	7 (3.3%)	1 (1.6%)		8 (2.9%)	
I	112	53 (29.9%)	45 (21.0%)	14 (21.9%)		59 (21.2%)	
II	214	76 (42.9%)	104 (48.6%)	34 (53.1%)		138 (49.6%)	
III–IV	115	42 (23.7%)	58 (27.1%)	15 (23.4%)	0.458	73 (26.3%)	0.192
Tumor size (cm)							
≤ 2	178	74 (42.8%)	75 (35.5%)	29 (47.5%)		104 (38.2%)	
> 2	267	99 (57.2%)	136 (64.5%)	32 (52.5%)	0.154	168 (61.8%)	0.341
Bloom-Richardson grade							
1 or 2	311	124 (72.9%)	138 (68.7%)	49 (75.4%)		187 (70.3%)	
3	125	46 (27.1%)	63 (31.3%)	16 (24.6%)	0.487	79 (29.7%)	0.552
LN involvement							
Negative	275	106 (56.1%)	128 (56.4%)	41 (56.2%)		169 (56.3%)	
Positive	214	83 (43.9%)	99 (43.6%)	32 (43.8%)	0.998	131 (43.7%)	0.957
ER							
Negative	190	72 (37.3%)	87 (37.0%)	31 (41.9%)		118 (38.2%)	
Positive	312	121 (62.7%)	148 (63.0%)	43 (58.1%)	0.738	191 (61.8%)	0.843
PR							
Negative	227	90 (46.6%)	105 (44.7%)	32 (43.2%)		137 (44.3%)	
Positive	275	103 (53.4%)	130 (55.3%)	42 (56.8%)	0.861	172 (55.7%)	0.615
HER2							
Negative	361	135 (88.8%)	176 (97.2%)	50 (87.7%)		226 (95.0%)	
Positive	29	17 (11.2%)	5 (2.8%)	7 (12.3%)	0.005	12 (5.0%)	0.024
Ki67							
≤ 14%	191	79 (40.9%)	89 (37.9%)	23 (31.1%)		112 (36.2%)	
> 14%	311	114 (59.1%)	146 (62.1%)	51 (68.9%)	0.332	197 (63.8%)	0.293
P53							
Negative	389	158 (81.9%)	174 (74.0%)	57 (77.0%)		231 (74.8%)	
Positive	113	35 (18.1%)	61 (26.0%)	17 (23.0%)	0.155	78 (25.2%)	0.064

Supplementary Table 6: Associations between rs4790264 and the clinicopathological features

clinicopathological features	NO.	TT (%)	TG (%)	GG (%)	*p* value	TG + GG (%)	*p* value
Clinic stage(UICC)							
0	14	8 (3.0%)	6 (3.5%)	0 (0.0%)		6 (3.2%)	
I	112	77 (28.6%)	32 (18.5%)	3 (23.1%)		35 (18.8%)	
II	214	121 (45.0%)	85 (49.1%)	8 (61.5%)		93 (50.0%)	
III–IV	115	63 (23.4%)	50 (28.9%)	2 (15.4%)	0.265	52 (28.0%)	0.122
Tumor size (cm)							
≤ 2	178	107 (40.7%)	67 (39.6%)	4 (30.8%)		71 (39.0%)	
> 2	267	156 (59.3%)	102 (60.4%)	9 (69.2%)	0.770	111 (61.0%)	0.723
Bloom-Richardson grade							
1 or 2	311	177 (69.1%)	119 (72.6%)	15 (93.8%)		134 (74.4%)	
3	125	79 (30.9%)	45 (27.4%)	1 (6.2%)	0.098	46 (25.6%)	0.228
LN involvement							
Negative	275	169 (58.3%)	93 (51.1%)	13 (76.5%)		106 (53.3%)	
Positive	214	121 (41.7%)	89 (48.9%)	4 (23.5%)	0.072	93 (46.7%)	0.273
ER							
Negative	190	112 (37.6%)	68 (36.6%)	10 (55.6%)		78 (38.2%)	
Positive	312	186 (62.4%)	118 (63.4%)	8 (44.4%)	0.281	126 (61.8%)	0.883
PR							
Negative	227	134 (45.0%)	82 (44.1%)	11 (61.1%)		93 (45.6%)	
Positive	275	164 (55.0%)	104 (55.9%)	7 (38.9%)	0.379	111 (54.4%)	0.891
HER2							
Negative	361	218 (92.0%)	129 (94.2%)	14 (87.5%)		143 (93.5%)	
Positive	29	19 (8.0%)	8 (5.8%)	2 (12.5%)	0.543	10 (6.5%)	0.586
Ki67							
≤ 14%	191	118 (39.6%)	68 (36.6%)	5 (27.8%)		73 (35.8%)	
> 14%	311	180 (60.4%)	118 (63.4%)	13 (72.2%)	0.526	131 (64.2%)	0.387
P53							
Negative	389	234 (78.5%)	142 (76.3%)	13 (72.2%)		155 (76.0%)	
Positive	113	64 (21.5%)	44 (23.7%)	5 (27.8%)	0.738	49 (24.0%)	0.503

Supplementary Table 7: Associations between rs8072363 and the clinicopathological features

clinicopathological features	NO.	TT (%)	TC (%)	CC (%)	*p* value	TC + CC (%)	*p* value
Clinic stage(UICC)							
0	14	8 (3.0%)	6 (3.5%)	0 (0.0%)		6 (3.2%)	
I	112	77 (28.6%)	32 (18.5%)	3 (23.1%)		35 (18.8%)	
II	214	120 (44.6%)	86 (49.7%)	8 (61.5%)		94 (50.5%)	
III–IV	115	64 (23.8%)	49 (28.3%)	2 (15.4%)	0.281	51 (27.4%)	0.126
Tumor size (cm)							
≤ 2	178	107 (40.7%)	67 (39.6%)	4 (30.8%)		71 (39.0%)	
> 2	267	156 (59.3%)	102 (60.4%)	9 (69.2%)	0.770	111 (61.0%)	0.723
Bloom-Richardson grade							
1 or 2	311	177 (69.1%)	119 (72.6%)	15 (93.8%)		134 (74.4%)	
3	125	79 (30.9%)	45 (27.4%)	1 (6.2%)	0.098	46 (25.6%)	0.228
LN involvement							
Negative	275	169 (58.1%)	93 (51.4%)	13 (76.5%)		106 (53.5%)	
Positive	214	122 (41.9%)	88 (48.6%)	4 (23.5%)	0.084	92 (46.5%)	0.320
ER							
Negative	190	112 (37.6%)	68 (36.6%)	10 (55.6%)		78 (38.2%)	
Positive	312	186 (62.4%)	118 (63.4%)	8 (44.4%)	0.281	126 (61.8%)	0.883
PR							
Negative	227	134 (45.0%)	82 (44.1%)	11 (61.1%)		93 (45.6%)	
Positive	275	164 (55.0%)	104 (55.9%)	7 (38.9%)	0.379	111 (54.4%)	0.891
HER2							
Negative	361	217 (91.6%)	130 (94.9%)	14 (87.5%)		144 (94.1%)	
Positive	29	20 (8.4%)	7 (5.1%)	2 (12.5%)	0.364	9 (5.9%)	0.347
Ki67							
≤ 14%	191	117 (39.3%)	69 (37.1%)	5 (27.8%)		74 (36.3%)	
> 14%	311	181 (60.7%)	117 (62.9%)	13 (72.2%)	0.588	130 (63.7%)	0.498
P53							
Negative	389	233 (78.2%)	143 (76.9%)	13 (72.2%)		156 (76.5%)	
Positive	113	65 (21.8%)	43 (23.1%)	5 (27.8%)	0.815	48 (23.5%)	0.651

The reaction conditions and protocol for the genotyping

Genomic DNA was isolated from EDTA-anticoagulated whole blood using AxyPrep Blood Genomic DNA Miniprep Kit (Axygen Biotechnology, US). The SNaPshot SNP assay was carried out to detect the dimorphism of the seven SNPs loci. PCR and extension primers were designed with Primer3 Online. PCR reaction system included 1× HotStarTaq buffer, 3.0 mM Mg^{2+}, 0.3 mM dNTP, 1 µl multiplex PCR primers(each at a final concentration of 1 µM) ,1 U HotStarTaq polymerase (Qiagen Inc.) and 1 µl templates DNA(5–10 ng/µl) in a total volume of 20 µl. The cycling program was as follows: denaturation at 95ºC for 15 min followed by 11 cycles of 94ºC for 20 sec, 65ºC–0.5ºC per cycle for 40 sec, 72ºC for 90 sec and 24 cycles of 94ºC for 20 sec, 59ºC for 30 sec, 72ºC for 90 sec; then an additional extension at 72ºC for 2 min. Thereafter, 1 U SAP (Promega) and 1 U ExoI (Epicentre) were added to 10 µl of PCR product for purification. The mixture was incubated at 37ºC for 60 min, followed by incubation at 75ºC for 15 min. The minisequencing reaction was performed using the SNaPshot™ kit (Applied Biosystems) with 5µl of SNaPshot ready reaction mix, 1 µl mixture of extension primers(each at a final concentration of 0.8 µM) and 2 µl of purified multiplex PCR products in a 10 µl total volume. The cycling program was as follows: denaturation at 96ºC for 60 sec followed by 28 cycles of 96ºC for 10 sec, 50ºC for 5 sec, 60ºC for 30 sec. Then, 10 µl of extension products were purified by 60min incubation with 1 U SAP (Promega) at 37ºC and a subsequent 15 min incubation at 80ºC. The minisequencing products (0.5µl) were mixed with 9 µl of HiDi™ formamide(Applied Biosystems) and 0.5 µl of GeneScan-120 LIZ Size Standard (Applied Biosystems) and denatured at 95°C for 5 minutes. The Minisequencing products were loaded onto an ABI3130XL Genetic Analyzer. The resulting data was analyzed with GeneMapper™ 4.1 Software (Applied Biosystems).

Supplementary Table 8: PCR primer sequences

SNPs	PCR primers
rs1050390	Forward:5′-TTCCCAGGGCAGTTGTTTGAAG-3′<br>Reverse:5′-CTGAAGCTAGACATGTGCTTTGAAATG-3′
rs1050461	Forward:5′-TTCCCAGGGCAGTTGTTTGAAG-3′<br>Reverse:5′-CTGAAGCTAGACATGTGCTTTGAAATG-3′
rs2285747	F:5′-TGAAGGGTTCTCCAGGACAAGCTA-3′<br>Reverse:5′-AAGCAGGGTGCCAACAGAAGTG-3′
rs2472614	Forward:5′-TTAAAAACCTGGAAGATGCCAAATG-3′<br>Reverse:5′-TGTAGTAAGGGCTGCCATCAGAGG-3′
rs3786054	Forward:5′-TCACAAAGCAGGCCAGACACAC-3′<br>Reverse:5′-GAAAGGTGGCTTCCTGGCAGA-3′
rs4790264	Forward:5′-TGAAGGGTTCTCCAGGACAAGCTA-3′<br>Reverse:5′-AAGCAGGGTGCCAACAGAAGTG-3′
rs8072363	Forward:5′-TTCCCAGGGCAGTTGTTTGAAG-3′<br>Reverse:5′-CTGAAGCTAGACATGTGCTTTGAAATG-3′

Extension primer sequences

SNPs	extension primers
rs1050390	SR: TTTTTTTTTCATCTGTTCTTGGTCTTTTGTGAC
rs1050461	SR: TATCATGGGGGAAAAAATACCA
rs2285747	SR: TTTTTTTTTTTTTTTTTTTTTTTTCTGAACAGAGATCCTTGATCCAG
rs2472614	SR: TTTTTTTTTTTTTTTTGATGATGGGGGAGCTGACTTA
rs3786054	SR: TTTTTTTTTTTTTTTTTTTTTTTTTTTTTTTAGGCACCATTCCACACAAGA
rs4790264	SR: TTTTTTTTTTTTTTTTTTTTTTTTAGTGGCCCCCTTTCTTCCC
rs8072363	SR: AACTACACTACTGAACTAATTGAAACTGGA
